# Editorial: Regulation and coordination of the different DNA damage responses and their role in tissue homeostasis maintenance

**DOI:** 10.3389/fcell.2023.1175155

**Published:** 2023-03-13

**Authors:** Luis Alberto Baena-López, Antonio Baonza, Carlos Estella, Héctor Herranz

**Affiliations:** ^1^ Sir William Dunn School of Pathology, University of Oxford, Oxford, United Kingdom; ^2^ Centro de Biología Molecular “Severo Ochoa”, CSIC-UAM, Madrid, Spain; ^3^ Department of Cellular and Molecular Medicine, University of Copenhagen, Copenhagen, Denmark

**Keywords:** DNA damage, apoptosis, cell proliferation, tissue homeostasis, DNA damage response

Cells are constantly exposed to multiple internal and external stressors that can damage their genetic material. To maintain genome stability and integrity, cells have evolved sophisticated molecular DNA damage response pathways (DDRs), that detect and repair DNA lesions. The activation of these pathways triggers a wide range of cellular responses, including cell cycle arrest, DNA repair, senescence, and apoptosis, which prevent the proliferation of cells with potentially harmful DNA changes. Depending on the cell type, stage of development, and proliferation status, these “life” vs. “death” decisions can differ. Apoptosis, in particular, is of special interest as defects in its induction can contribute to tumorigenesis or the resistance of cancer cells to therapeutic agents such as radiotherapy. Although much progress has been made in our comprehension of the factors that form the DDR pathways and their activation, much remains to be investigated on the coordination and integration of their induced set of cellular responses. Addressing these fundamental biological questions is crucial to fully understand the molecular basis maintaining tissue homeostasis upon DNA damage as well as preventing the formation of tumours. This special issue was envisioned to provide new insights regarding this topic while illustrating some of the complexities of the field. The Research Topic includes several literature reviews, as well as two original articles that present new findings. We hope that these articles contribute to picture our current understanding about how the DDR coordinates the induction of numerous cellular events while stimulating new lines of future research in this important area of biology.

Intracellular and extracellular signals can modulate numerous cellular events after DNA damage. For example, the Notch pathway, which is involved in a variety of developmental and physiological processes, can regulate the apoptotic response in cells showing lesions on their DNA. The Notch receptor negatively regulates the DDR by attenuating the activity of the phosphatidylinositol-3-like protein kinase ataxia-telengiectasia mutated (ATM) (Vermezovic et al., 2015). In this way, Notch1 inhibition increases ATM-dependent apoptosis in response to DNA damage. In a new study conducted by Neetu Saini and colleagues, they explore the role of the nucleolar localization of the Notch1 intracellular domain (NIC1) during the induced apoptotic response by DNA damage (Saini et al., 2022). They specifically discovered that NIC1 can interact with a molecular cascade in the nucleolus to prevent DNA damage-dependent apoptosis. Through targeted genetic mutations, they also show that the subcellular localization of NIC1 in the nucleolus is regulated by a protein called Sirtuin1. These findings, along with previous research from the same laboratory, shed new light on the role of Notch signalling in regulating the cellular response to DNA damage (Saini and Sarin, 2020).

The proteins involved in the cell cycle control regulate the precise duplication and propagation of genomes. Deregulation of cell cycle can affect genome stability and consequently tissue growth. Cyclins are key cell cycle regulators that timely activate cyclin-dependent kinases at different stages of the cell cycle. Cyclin E promotes G1-S progression in proliferating cells. Cyclin E upregulation above physiological levels can result in replication stress and genomic instability (Fagundes and Teixeira, 2021). Not surprisingly, Cyclin E is commonly amplified in human cancers (Macheret and Halazonetis, 2015). Although the roles of Cyclin E in normal proliferating cells have been extensively studied, the implications of Cyclin E deregulation in non-proliferating tissues remain poorly understood. In a study presented in this Research Topic, Molano-Fernández and colleagues use the accessory gland of *Drosophila* as an *in vivo* model to study the consequences of Cyclin E upregulation in non-proliferating cells (Molano-Fernández et al., 2023). The accessory gland of the fruit fly is the functional orthologue of the human prostate and is emerging as a useful platform to model different aspects related to prostate cancer (Rambur et al., 2021). Molano-Fernández and colleagues show that Cyclin E induces variable levels of endoreplication, a singular type of cycle in which cells go through rounds of DNA replication in the absence of cell division. A direct consequence of endoreplication is an increased cellular ploidy. Cyclin E-induced endoreplication in the accessory gland is associated with extensive DNA damage and defects in size and cellular organization.

The activation of the DDR commonly induces cell proliferation arrest and apoptosis. However, these cellular outcomes vary depending on cell type and context and can impact not only cell survival but also the efficacy of therapeutic strategies for different diseases, including cancer. For example, ionizing radiation is particularly effective in targeting proliferative cells (Ruiz-Losada et al., 2022). In the context of radiation-induced DNA damage, Baonza and colleagues describe the cascade of cellular responses triggered by this type of damage in several *Drosophila* tissues and their interplay with the apoptosis machinery. The authors also explore hypothetical intrinsic mechanisms that could mitigate the apoptotic pathway in response to radiation-induced DNA damage, and how these mechanisms could impact the responsiveness of transformed cells to radiation therapy (Baonza et al., 2022).

The activation of apoptosis upon DNA damage seems to coordinate not only the elimination of defective cells but also the regeneration response in healthy surrounding cells. This coordination is crucial to maintain tissue homeostasis. Serras provides an interesting review describing the potential function of the MAP3 kinase Ask1 as a signaling coordinator of both cell death and regeneration induced by DDR (Serras, 2022). He describes how cells with DNA damage produce large amounts of reactive oxygen species (ROS), which can activate Ask1, JNK-signalling and ultimately lead to apoptosis. This process eliminates damaged cells and could seemingly coordinate the regenerative response in healthy surrounding cells. In this regard, the author hypothesise that a wave of ROS from apoptotic cells and a moderate activation of Ask1 in healthy cells can facilitate the regenerative process. This opens new avenues for future research in the regeneration field.

The behaviour of cells in both developmental and pathological contexts is determined by their unique gene expression profile. While changes in DNA sequence caused by mutagenic agents is irreversible, epigenetic modifications can reversibly affect gene expression. DNA methylation, particularly in CpG-enriched sequences, was the first recognized epigenetic modification, but recent research has revealed that the methylation status of various RNA types also significantly affects their structural and functional characteristics. In their literature review, Alagia and Gullerova provide a comprehensive overview of our current knowledge regarding the molecular factors contributing to epigenetic regulation *via* RNA methylation. They also describe how innovative techniques are facilitating the study of these crucial modifications, and ultimately the physiological and pathological implications, mainly in tumours, of these epigenetic changes (Alagia and Gullerova, 2022). Although many aspects of the methylation and demethylation process have been elucidated in recent years, the manuscript identifies several critical questions that remain unresolved. Addressing these questions could pave the way for developing targeted therapies with significant clinical implications.
